# Macrophage barrier in the tumor microenvironment and potential clinical applications

**DOI:** 10.1186/s12964-023-01424-6

**Published:** 2024-01-26

**Authors:** Shuai Ji, Yuqing Shi, Bo Yin

**Affiliations:** 1 Department of Urinary Surgery, The Shengjing Hospital of China Medical University, Shenyang, 110022 China; 2 Department of Respiratory Medicine, Shenyang 10th People’s Hospital, Shenyang, 110096 China

**Keywords:** Macrophage, Tumor-associated macrophages (TAMs), Tumor microenvironment, Tumor immunology

## Abstract

The tumor microenvironment (TME) constitutes a complex microenvironment comprising a diverse array of immune cells and stromal components. Within this intricate context, tumor-associated macrophages (TAMs) exhibit notable spatial heterogeneity. This heterogeneity contributes to various facets of tumor behavior, including immune response modulation, angiogenesis, tissue remodeling, and metastatic potential. This review summarizes the spatial distribution of macrophages in both the physiological environment and the TME. Moreover, this paper explores the intricate interactions between TAMs and diverse immune cell populations (T cells, dendritic cells, neutrophils, natural killer cells, and other immune cells) within the TME. These bidirectional exchanges form a complex network of immune interactions that influence tumor immune surveillance and evasion strategies. Investigating TAM heterogeneity and its intricate interactions with different immune cell populations offers potential avenues for therapeutic interventions. Additionally, this paper discusses therapeutic strategies targeting macrophages, aiming to uncover novel approaches for immunotherapy.

Video Abstract

**Supplementary Information:**

The online version contains supplementary material available at 10.1186/s12964-023-01424-6.

## Introduce

 The tissues of all mammals are infiltrated by macrophages, which co-develop with the different organs they inhabit, and to some extent, these macrophages maintain their numbers and phenotypes until the environment changes. However, the vast majority of macrophages in almost all tissues are divided into three sources: blood/bone marrow derived monocytes, tissue resident macrophages and bone marrow derived inhibitory cells (MDSCs) [1, 2].These macrophages have typical phagocyt-related immune sentinel and clearance functions, and can be well adapted to the tissues they live in, so that macrophages can play the function of maintaining tissue or organ homeostasis, which brings about the cognition that “macrophages are not only immune cells, but microenvironment equilibrators“ [3–5]. However, when the homeostasis is broken, namely when inflammation, injury and canceration occur, the macrophage pool in the tissue will change, in terms of quantity, phenotype, source and proportion of different sources, especially the macrophage subsets that help the occurrence and development of tumor appear in the tumor tissue [5]. These macrophage subgroups play a role in inhibiting immune killing, promoting immune escape of tumor cells and malignant proliferation and metastasis.

Although the research on macrophages in tumors has lasted for nearly a century [6], we only really began to study the significance of macrophages in tumor occurrence and development in the 1970s [7], and gradually revealed the phenotypic heterogeneity, functional heterogeneity and source heterogeneity of macrophage subpopulation [1]. In this paper, we continue our understanding of the progressive immunosuppressive phenotype of macrophages in the TME and, based on this, explore the spatial barrier that macrophages form around tumor cells. At the same time, we summarize the influence of macrophages on other stromal cells in the TME, and speculate that the formation of macrophage barrier at the early stage of the tumor is one of the important reasons for the malignant progression of the tumor.

## Spatial distribution of macrophages under physiological conditions

Macrophages are distributed in all tissues of the human body, including brain, peritoneum, muscle, lung, epidermis and liver. In the physiological state, macrophages exhibit a regular spatial arrangement, so that each macrophage occupies its own inch of territory [8, 9]. The spatial distribution of macrophages can be attributed to the contact inhibition mechanism inherent in cells, which leads to the mutual repulsion of macrophages [8, 10]. In normal tissues, the death of a macrophage will inevitably lead to the formation of a blank area, and at the same time cause the loss of the rejection effect of this area on other macrophages, thus triggering the proliferation of neighboring macrophages, which will supplement the blank area and balance the rejection effect once again. However, the territorial effects of contact inhibition and rejection cannot fully explain all macrophage spatial distribution patterns [11, 12]. For example, in vitro culture model, ThP-1-derived macrophages showed clump growth phenomenon, and macrophages were densely arranged in the splenic red pulp and the subcapsular sinuses of lymph nodes [11, 12]. Guilliams et al. introduced culture scaffolds to enrich the mechanism of different special spatial distribution states of macrophages, namely interleukin 34(IL-34),The source of colony stimulating factor 1([CSF1], also known as M-CSF) and CSF2(or GM-CSF), which maintain the growth and development of macrophages, leads to the chemotaxis of macrophages to different positions [13]. Of course, the dense arrangement and/or regular distribution of macrophages under physiological conditions is similar to the distribution of police stations in human society, so as to facilitate the monitoring of restless molecules in the entire environment, and also realize the rapid “alarm”.

## The spatial distribution of macrophages in the TME is the basis of the formation of macrophage barrier

The spatial distribution of macrophages in physiological state determines that macrophages have a good role in immune surveillance. Thus, in the mouse YAP^+^ hepatocellular carcinoma model, macrophages have rapidly gathered around the tumor cells while the liver cancer cells are still in the single-cell state (tumor-initiating TIC cells) [14]. In mouse non-small cell lung cancer, alveolar macrophages localize near tumor cells after inoculation and enhance their antigen presentation and tissue remodeling program in response to tumor signals [15]. Of course, the rapid aggregation of macrophages comes from the recruitment effect of CSF1 on macrophages, so the secretion and activity of CSF1 determine the spatial distribution of macrophages to a certain extent. CSF1 in the TME is mainly derived from tumor cells and tumor-associated fibroblasts (TAFs), and studies based on spatial transcriptomics have also confirmed the co-localization of macrophages with these two types of cells [16–18]. As the tumor progresses, the tumor volume gradually increases, resulting in a relative “macrophage-free void” between tumor cells and tumor fibroblasts. Due to the lack of CSF1 consumption and the lack of contact-inhibited rejection, this void becomes a suitable niche for the survival of macrophages [19]. Although this relative blank area is spread throughout the tumor tissue, the distribution of cells is not absolutely uniform, because in addition to CSF1, CCL2, CCL3, CCL4, CCL5, CCL20, CCL18 and CSF-2 may play the role of recruitment to macrophages, and due to the difference of action threshold and cytokine concentration [20]. The spatial distribution of macrophages is also quite different. And more research is needed to determine the exact mechanism.

In the TME, tumor-associated macrophages (TAMs) that inhibit angiogenesis and activate anti-tumor immunity are defined as M1 TAMs, and TAMs that promote tumor growth, invasion, and metastasis are defined as M2 TAMs [21]. The spatial distribution of M1 TAMs and M2 TAMs is also different. In the non-small cell lung cancer model, M2 TAMs were mostly clustered in the peripheral region of the tumor and were closer to the tumor cells [22]. In the study of gastric cancer, the researchers found that CD68^+^IRF8^+^M1 TAMs and CD68^+^CD206^+^M2 TAMs were closest to the tumor cells, while CD68^+^CD163^+^CD206^+^M2 TAMs were farthest from the tumor cells [23]. In addition, the CD206 expression of CD68^+^CD206^+^M2 TAMs closer to the tumor cells was lower, while the expression of CD163 and CD206 on TAMs was negatively correlated [23]. Consistently, in the pancreatic cancer model, CD86^+^IRF5^+^M1 TAMs were closer to the tumor cells than CD163^+^CD206^+^M2 TAMs [24]. The proximity of CD163^+^CD206^+^M2 TAMs to tumor cells is closely related to the survival rate of patients [23, 24]. This suggests that macrophages at different spatial locations have different functional phenotypes, which may affect prognosis. For example, in a sample of adult diffuse gliomas, the histological properties of isocitrate dehydrogenase wild-type glioblastoma (GBM-IDH-WT, grade 4) indicate microangiogenesis and necrosis, with IBA1^+^/CXCL3^+^TAMs located primarily in the perinecrotic area and IBA1^+^/TMEM119^+^TAMs located near microvessels [25]. IBA1^+^/TREM2^+^TAMs were diffusely distributed in the parenchyma of GBM [25]. In a breast cancer brain metastases model, HR^+^/HER2^−^BM compared to other BC subtypes showed a higher density of CD68^+^ microglia/macrophages in the tumor area and a shorter distance between PD-L1^+^CK^+^ tumor cells and PD-1^+^CD3^+^T lymphocytes [26]. And PD-L1^+^CD163^+^M2 polarized microglia/macrophages and PD-1^+^CD3^+^T lymphocytes in the stroma at a shorter distance [26]. The short distance between PD-L1^+^CD163^+^M2 TAMs and PD-1^+^CD3^+^T lymphocytes was also associated with a shortened survival period.

The infiltration of NK cells, CD56^+^NKT cells, and CD56^+^NKp46^+^NKT cells in malignant tissues was significantly lower than that in benign tissues, while the infiltration of CD68^+^ macrophages, iDC, pDC, and CD123^+^CD15^+^ granulocytes in malignant tissues was significantly higher than that in benign tissues [27]. Meanwhile, NK cells, iDC, mDC, and CD123^+^CD15^+^ granulocytes were more infiltrated in the tumor area, while all macrophage populations and CD56^+^NKT and NKp46^+^NKT cells were more infiltrated in the stroma [27]. The macrophages in this study tended to be distributed within the peripheral stroma of malignant tumors, which is consistent with the findings in gastric and pancreatic cancer [23, 24, 27]. In the rectal cancer model, SPP1^+^ macrophages and FAP^+^ fibroblasts co-locate within the tumor, where they contribute to the formation of connective tissue and prevent the invasion of the tumor core by T cells or B cells [18].And the LGALS9-CD44/CD45 inhibitory signal displayed by macrophages may inhibit the activation of T and B lymphocytes that are spatially adjacent to macrophages [28]. These findings have led to the idea that the aggregation of macrophages in the peripheral stroma of the tumor may be an important manifestation and condition for malignant progression of the tumor, and that this aggregation constitutes a spatial barrier of macrophages that inhibits tumor immunity and promotes tumor progression (Table 1).

**Table 1:** Spatial heterogeneity of macrophages in the TME

Cancer type	Model source	Technology	Spatial distribution	Ref.
Breast cancer	Human	Visium, scRNA-seq	Regions of elevated type I interferon within tumors exhibit an enrichment of CXCL10^+^ TAMs, which interact with T cells.	[29]
Hepatocellular carcinoma	Human	Visium	The central tumor core shows significant upregulation of CCL15 expression, attracting and polarizing M2-like macrophages.	[30]
Melanoma	Human	DSP, PickSeq, CyCIF	Macrophages with high PDL1 expression infiltrate the invasive tumor border, inhibiting immune cytotoxicity by engaging with PD1^+^ CTLs.	[31]
Neuroblastoma	Alk^F1178L^; TH-MYCN or Alk^Y1282S^; TH-MYCN mice	Visium, scRNA-seq, TCR repertoire	Co-localization of CD4^+^ T cells and macrophages.	[32]
Gliomas	Human	ISH, scRNA-seq, WES	Blood-derived TAMs significantly infiltrate pre-treated gliomas, congregating around blood vessels and necrotic areas.	[33]
Colorectal cancer	Human	Visium, scRNA-seq	Interaction between FAP^+^ fibroblasts and SPP1^+^ macrophages occurs in CRC. Their coexistence is linked to extracellular matrix expression.	[18]
Non-small cell lung carcinoma	Mice C57BL/6, Ms4a3-tdTom reporter and CD169-DT; Human	scRNA-seq, scATAC-seq	Tissue-resident macrophages accumulate near tumor cells during the early stages of tumor formation.	[15]

## Macrophages form a spatial barrier to prevent immune killing

### Macrophage and CD8^+^T cells

CD8^+^T cells have the ability to selectively recognize and kill cancer cells due to specific responses to tumor-expressed antigens, including tumor-specific (mutants and viruses) neoantigens and autoantigens (also known as tumor-associated or shared antigens) [34–41]. However, even when such CD8^+^T cells are found in cancer patients with a specific response to tumor antigens, tumors expressing highly immunogenic neoantigens often do not stop progressing [42, 43]. The coexistence of sustained tumor growth and T-cell infiltration was described as early as 1968 by Ingegerd and Karl Erik Hellstrom et al., and is now known as the “Hellstrom paradox”, which partly explains the dysfunction of tumor-reactive CD8^+^T cells during tumorigenesis and progression [44, 45]. We have learned from studies of CD8^+^T cells isolated from progressive tumors that CD8^+^T cell dysfunction includes tumor-infiltrating lymphocytes (TIL) expressing a variety of inhibitory receptors (such as PD1, LAG3, CTLA4, and TIM3),Reduced or non-production of cytokines (such as interferon-gamma and TNF) or cytotoxic molecules (such as granulozyme and perforin) [46–48]. These dysfunctions, such as “incompetence”, “tolerance” and “exhaustion”, are the main manifestation of T cell function in low reactivity.

In mouse models, alveolar macrophages evolved from “scattered around lung cancer cells” to “clustered around tumor foci”, creating a spatial barrier of macrophages around the tumor [15]. This barrier also induces the EMT program to promote cell invasion while promoting the Treg cell response, protecting tumor cells from killing by CD8^+^T cells [15]. In the model of melanoma lymph node metastasis, the spatial distribution of macrophages presents two states, one is limited to the periphery of the tumor, the other is limited to the internal specific areas of the tumor [16]. In addition, CD68^+^ macrophages were farthest from melanoma cells in brain metastases, but closer in lung and liver metastases. Of course, CD8^+^T cells were significantly depleted in the melanoma area regardless of the macrophage distribution [16].

Both infiltration and exhaustion of CD8^+^T cells were positively correlated with macrophage infiltration [27, 49, 50]. For example, in a urothelium carcinoma model, macrophages express PDCD1LG2, CD274, CD80, and CD86, which inhibit the activation of CD8^+^T cells in response to immune checkpoints CTLA-4 and PDCD1. The ratio of macrophages to CD8^+^T cells is positively correlated, but macrophages are also positively correlated with CD8^+^T cell exhaustion [49]. In addition, compared with neighboring tissues, CTLA4^+^CD8^+^T, SPP1^+^ macrophages and MRC1(CD206)^+^ CCL18^+^ macrophages were also enriched in tumor tissues in the samples of colorectal cancer liver metastasis [50]. This makes it easier for macrophages to achieve inhibition of CD8^+^T tumor killing function. In mouse models of melanoma (B78ChOVA and B16ChOVA) and spontaneous breast cancer (MMTV-PyMTChOVA), rapid exhaustion of TAMs resulted in decreased expression of exhaustion markers PD-1, CD38 and TOX on tumor-infiltrating CD44^+^OT-ICD8^+^T cells. But CD44^+^OT-ICD8^+^T cells produced higher levels of IFN-γ and TNF-α, and slightly reduced tumor volume [51]. The exhaustion of CD8^+^T cells may be attributed to their co-localization with TAMs in spatial distribution [51, 52]. For example, after adoptive transfer of OT-I and P14 CD8^+^T(CD8^+^T cells labeled with P14 gene are suitable for the study of T cell colonization and tracing) cells in B78ChoVa-carrying mice, researchers found that (after enzymatic digestion) the proportion of T-T cell complex and the proportion of TAM-T cell complex formed by OT-ICD8^+^T via antigen-specific synapses was significantly higher than that of P14 or endogenous CD8^+^T cells [51]. This phenomenon seems to partly explain the poor efficacy of CAR T cells in solid tumors. A study in metastatic melanoma models showed that cytotoxic T cells near the tumor edge and macrophages had the most obvious exhaustion, and macrophages near the tumor edge and near cytotoxic T cells also expressed more PD-L1 [53].The greatest exhaustion of cytotoxic T cells occurs in the area of the macrophage barrier, where macrophages are abundant and have more opportunity to come into contact with T cells within the “effective interaction distance” (considered by most studies to be the effective interaction distance within a radius of less than 20 μm).

Macrophages can effectively take up antigen fragments and interact with T cells within the “effective interaction distance” for a long time, and then cross-present tumor antigens to CD8^+^T cells through in vitro MHC-I/TCR signaling [54, 55]. Interestingly, some CD8^+^T cells also help macrophages downregulate their antigen presentation, making them better at becoming TAMs. For example, CD8^+^T cells in the depleted state of the inner region of the TME express CSF1 to promote the survival of monocytes/macrophages, so that when monocytes move to the interior of the microenvironment and gradually differentiate into TAMs, the antigenicity of macrophages will be down-regulated with the development of the depleted state of CD8^+^T cells [51]. In addition, in the melanoma mouse model, YTHDF2^+^ macrophages weakened their MHCI-like antigen cross-presentation function, thus reducing the activation and infiltration of CD8^+^T cells, while YTHDF2^+^ macrophages reduced the stability of their STAT1 mRNA, and were more likely to be induced into the M2 polarization phenotype [56]. This makes it possible to form a positive feedback loop between macrophages and CD8^+^T cells in depleted state to achieve immunosuppression. In addition, studies of primary glioblastoma and recurrent glioblastoma have found that bone marrow-derived macrophages tend to aggregate in the central region of primary glioblastoma and the peripheral region of recurrent glioblastoma [57]. In primary glioblastoma, there is more infiltration of CD8^+^T cells in recurrent glioblastoma than in primary glioblastoma, and these CD8^+^T cells are located in the central region of the tumor. These CD8^+^T cells have the typical rounded shape of resting T cells, and they rarely interact with CD68^+^ macrophages in the central region [57]. The reason for this phenomenon is most likely that CD8^+^T cells are taught by inhibitory macrophages in the peripheral region as they pass through the peripheral region, thus transforming into a depleted or resting state. For example, CD8^+^T cells are depleted by IL10 secreted by M2 TAMs [58].

Expression of immune-related receptors on macrophages is also associated with impaired T cell function, which is restored when the function of these receptors is suppressed. Aromatic hydrocarbon receptors (AhR), sensors of tryptophan metabolites and potent immune tuners, are highly expressed in TAMs of PDAC. Inhibition of AhR restored the inflammatory phenotype of macrophages and promoted the infiltration of IFNγ^+^CD8^+^T cells, which weakened Ahr-dependent tumor growth [59]. Inhibition of B7-H3 expression in highly serous ovarian cancer (HGSOC) also reverses macrophage inhibition of IFNγ^+^CD8^+^T cells [60]. Interleukin-15 plays an important antitumor role in tumor cell differentiation by inducing T immunotherapy and proliferation. However, in a mouse model of breast cancer, IL-15Rα^+^TAM releases IL-15Rc (the IL-15/IL-15Rα complex) to reduce the expression of chemokine CX3CL1 in tumor cells, thereby reducing recruitment to CD8^+^T cells [61]. Meanwhile, non-transcriptional activity of HIF-1α reduces CX3CL1 degradation, while IL-15Rc reduces both HIF-1α and CX3CL1 levels in tumor cells [61]. Thus, blocking IL-15Rc formation seems to restore CD8^+^T cell infiltration. However, HIF-1α, as a major regulatory factor in response to hypoxia conditions, promotes the immunosuppressive phenotype of macrophages, which may inhibit the function of CD8^+^T cells with increased infiltration, resulting in poor long-term therapeutic effect [22].

**Fig. 1:**
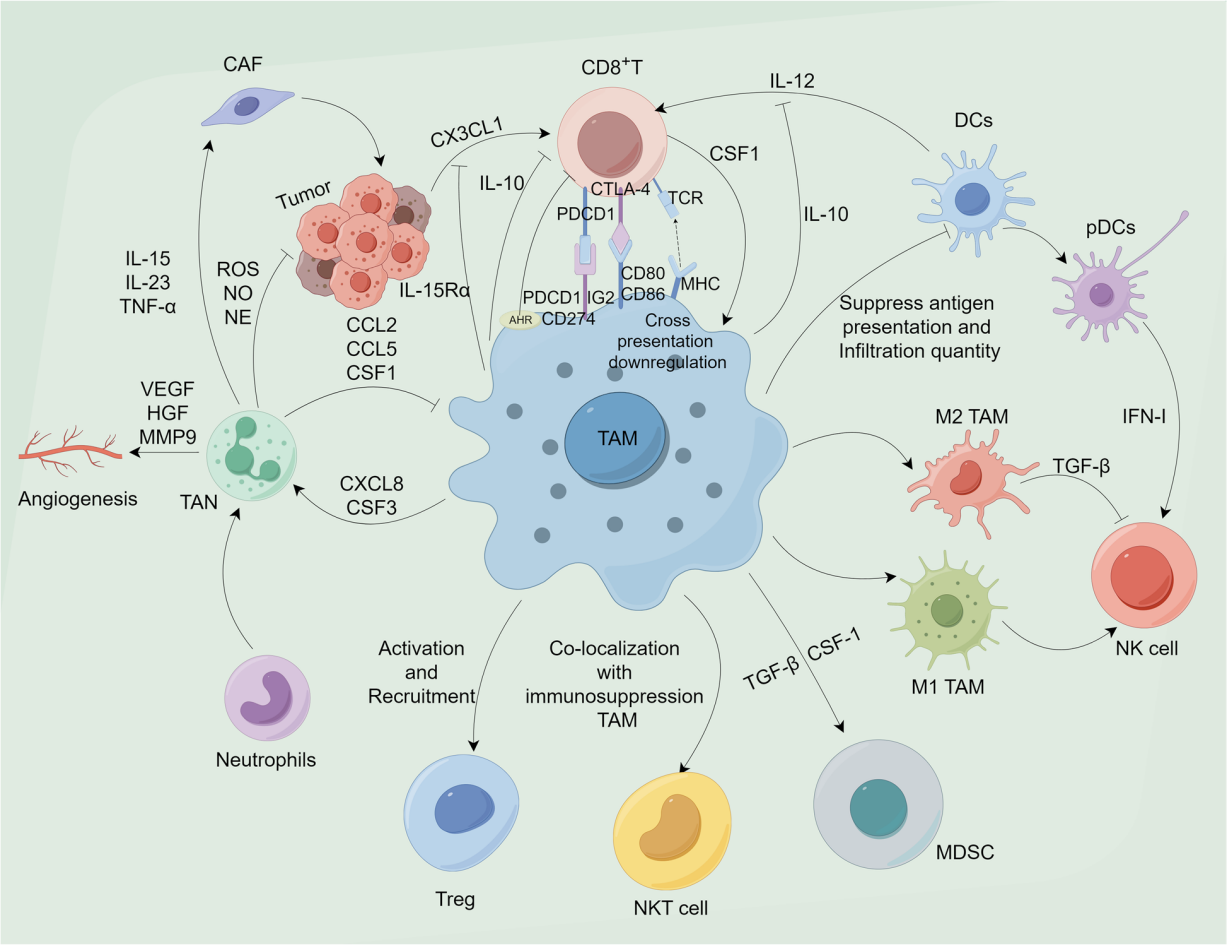
TAMs mediate immune cell regulation in TME. Tumor-associated macrophages (TAMs) intricately modulate the anti-tumor immune response within the tumor immune microenvironment through subtle interactions with distinct immune cell subsets. TAMs activate immune checkpoints, downregulate antigen presentation, and secrete regulatory factors to coordinate CD8 + T-cell responses. Additionally, TAMs suppress dendritic cell antigen presentation and infiltration. TAMs secrete TGF-β and CSF-1 can promote the amplification of MDSCs. Concurrently, TAMs recruit immune-suppressive Treg cells and inhibit the functions of NKT cells, leading to suppressive effects. By interacting with tumor-associated neutrophils, TAMs further facilitate tumor cell growth. Moreover, TAMs regulate NK cell activation and inhibition through distinct phenotypes. The diagram illustrates certain molecular mechanisms by which TAMs mediate immune cell regulation

In conclusion, the macrophage barrier weakens the tumor killing function of CD8^+^T cells by reducing the activation and infiltration of CD8^+^T cells, or by increasing the infiltration of CD8^+^T cells but depleting them [52, 56, 57, 62] (Fig. 1).

### Macrophages and CD4^+^T cells

In recent years, more and more attention has been paid to the role of CD4^+^T cells in anti-tumor immunity and the mechanism involved in tumor immune escape.CD4^+^T cells, like macrophages, are a highly heterogeneous cell population. Efficent CD4^+^T cells are usually present in the TME in the form of regulatory T cells (iTreg and nTreg) or different subtypes of conventional helper T cells (Th1, Th2, Th17, Th9, Th22) [63–70]. Their role in cancer immunity is still controversial due to heterogeneity. Since most cancer cells do not express MHCII class molecules (HLA-DR), they cannot be directly recognized by CD4^+^T cells. Instead, necrotic tumor cells or vesicles released by cancer cells ingested by tumor stromal cells mainly enter the classical MHCII (HLA-DR) processing pathway to achieve cross-presentation of tumor antigens [71]. Monocytes/macrophages are the most abundant MHCII positive cells in most solid tumors, making the participation of CD4^+^T cells in the immune recognition process partly dependent on the presence of macrophages. For example, in early non-small cell lung cancer lesions, TAMs up-regulate the expression of MHCII genes and present antigens to CD4^+^T cells [15]. Interestingly, one study found that activated macrophages promote CXCL13^+^CD4^+^T cell infiltration via CXCL9, CXCL10, CXCL11, and IL15. Increased infiltration of this subset of T cells is associated with increased survival in melanoma patients, but this antitumor effect is limited for the overall microenvironment [72]. In addition, CD4^+^ effector T cells cooperate with activated iNOS expressing tumor-killer monocytes and macrophages to coordinate leading distal inflammatory cell death to eliminate MHC deletion and non-responsive cancer cells [73]. Of course, this subset of CD4^+^ effector T cells is only a small subset of the CD4^+^T cell population, but this result also provides a new potential target for targeted therapy.

However, interactions between macrophages and CD4^+^T cells are not always so beneficial for eliminating tumor cells. For example, initial CD4^+^T cells induced by macrophages may differentiate into TREGs in cirrhosis, ultimately contributing to immunosuppressive TME and HCC formation [74]. In addition, the promoting effect of TAMs on the proliferation of Treg cells led to an increase in the ratio of Treg cells /CD8^+^T cells [15]. Of course, TAM in the central region of the tumor can also impair the T cell response by directly expressing PD-L1 on the membrane and recruiting invasive Tregs from the tumor outside the tissue into the TME [75] (Fig. 1). Studies of gastric cancer models have found that although CD4^+^FoxP3^−^T cells, CD4^+^FoxP3^−^CTLA-4 T cells, and CD4^+^FoxP3^−^PD-L1^−^T cells are close to tumor cells, However, the distance between CD68^+^CD163^−^HLA-DR^+^(M1) macrophages and tumor cells makes it difficult for them to perform the ideal antigen presentation function [76].In a model of head and neck squamous cell carcinoma, PD-1^+^ helper T cells colocalized with CD163^+^TAMs within tumor tissue, and this colocalization significantly shortened overall survival compared with other subpopulations [77].

### Macrophages and dendritic cells (DCs)

cDC and pDC are two classical types of dendritic cells [78–81]. Among them, cDC1s favored MHC-I cross-presentation on CD8^+^T cells due to its ability to capture specific antigens, while cDC2s favored MHC-II cross-presentation on CD4^+^T cells [79–81]. pDC is a DC subgroup that secretes a high level of type I IFN after stimulation by toll-like receptors (TLRs), which results in the existence of anti-tumor effects of pDC in TME. In vitro studies have demonstrated that properly activated pDC can activate T cells [82]. And in vivo studies have found that pDC produces an effective immune response to established tumors [83]. However, some studies have shown that pDC has negative immunomodulatory properties in the TME and is associated with poor clinical outcomes due to tumor tolerance to tumor suppression [84, 85]. This result was attributed to the deficiency of type I IFN in regulatory pDC, decreased expression of costimulatory molecules, and upregulated expression of IDO and PD-L1 [86–88]. In addition, studies on esophageal cancer have shown that PD-L1^+^DC and PD-L1^+^TAMs are mostly concentrated in the extra-tumor stroma, which is related to poor prognosis [89]. Meanwhile, this co-localization of macrophages and DC may result in the loss of antigen presentation function of DC cells or a decrease in the number of DC cell infiltration. In the B78ChOVA melanoma model, an increase in the invasive abundance of TAMs was accompanied by a decrease in the abundance of CD103^+^cDC1 and CD11b^+^cDC2 as the tumor progressed [51]. This may inhibit antigen presentation, making it difficult for CD4^+^T and CD8^+^T cells to activate successfully. For example, in breast cancer, TAM secretes IL-10 to inhibit CD103^+^DC production of IL-12, leading to T cell inhibition and reduced T cell activation [90] (Fig. 1). Unfortunately, although previous studies have preliminarily revealed that the interaction between TAMs and DCs is not conducive to the prognosis of tumors, there are few literatures on the interaction between TAMs and DCs, so that the deep mechanism is not fully revealed.

### Macrophages and neutrophils

Neutrophils are the first line of defense against inflammation and infection. They are absorbed into tissues under the influence of chemotaxis in the vasculature to play an infection-fighting role. However, the activation and function of neutrophils in the TME is influenced by a variety of stimuli and is not uniform. These neutrophils become tumor-associated neutrophils (TANs). Like TAM, there are two main polarizing types of TANs, namely N1TANs, which plays an anti-tumor role, and N2 TANs, which promotes tumor progression. Many studies have explored the possible antitumor mechanisms of TANs. TANs infiltrating around cancer cells achieve antitumor function through the expression of co-stimulatory receptors such as 4-1BBL, OX40L and CD86 and promote activation of active T cells and secretion of interferon γ(IFN-γ) [91]. Interestingly, IFN-γ stimulates the release of IL-18 from TANs, thereby activating NK cells [92]. At the same time, TANs can also secrete TNF-α, thus promoting the activation of DC and CD8^+^T cells [93, 94]. TANs itself can directly kill cancer cells by secreting cytotoxic substances such as ROS, nitric oxide (NO) and neutrophil elastase (NE), thus reducing tumor growth and metastasis [95, 96]. However, more findings suggest that TANs may accelerate tumor progression by promoting cancer cell proliferation, invasion, angiogenesis, and immunosuppression. For example, cytokines secreted by TANs such as IL-17, IL-23, and TNF-α activate the protein kinase B/p38(Akt/p38) pathway, which enables mesenchymal stem cells (MSC) to transform into TAFs and ultimately promote tumor cell proliferation and metastasis [97]. Like macrophages, TANs secrete VEGF, HGF, and MMP9 to promote angiogenesis while also making cancer cells more aggressive [96, 98, 99]. TANs can inhibit T cell activation by secreting arginine-1, reactive oxygen species and nitric oxide after being induced by G-CSF and TGF-β [73, 100].

Results based on intrahepatic cholangiocarcinoma showed that almost all CD66b^+^TANs were located adjacent to CD68^+^TAMs, and TANs and TAMs constituted small cell communities (clusters) in about two-thirds of the samples [101]. These Tans-Tams clusters significantly enhanced the proliferation, invasion, and colony formation of HuCCT1, RBE, and SG231 cells compared to TANs or TAMs alone, and also resulted in a higher incidence of lung metastatic tumors [101]. In terms of mechanism, OSM (oncostatin M), which is preferentially expressed by TANs, and IL-11, which is preferentially expressed by TAMs, jointly activate STAT3 signaling in ICC (intrahepatic cholangiocarcinoma) cells, thus achieving the tumor promoting effect [101]. In addition, TANs also expressed a series of chemokines such as CCL2, CCL5 and CSF1, which may achieve TAMs infiltration through recruitment of macrophages [101]. Similarly, chemokines related to TANs, such as CXCL8 and CSF3, are secreted by TAMs [101]. Interestingly, the secretion of CSF1 and CXCL8 was further increased after the Tans-Tams co-culture, suggesting that there may be a positive feedback loop between TANs and TAMs, making them highly overlapping spatially and highly synergistic functionally (Fig. 1).

### Macrophages and myeloid-derived suppressor cells (MDSCs)

MDSCs is the precursor of dc, macrophages and granulocytes from bone marrow. It is recruited to the tumor focus by chemokines such as CCL2 and CCL5 to exert the tumor immunosuppressive function and jointly form the immunosuppressive tumor myeloid microenvironment [102, 103]. In recent years, a study has shown that tam secretes TGF- β and has a positive feedback effect. Continuous exposure to TGF- β and CSF-1 can promote the amplification of MDSCs [104]. Interestingly, a recent study found that TAMs and MDSCs are co-located at the edge of colorectal cancer, and this edge is more aggressive [105]. A study of HCC also found that TAM and MDSC are located at the edge of the tumor, which is related to the functional inhibition of CD8^+^T cells [106]. In addition, flow cytometry analysis of TRAMP/MICB spontaneous prostate tumor model showed that the number of MDSCs in spleen and tumor infiltrating area was significantly correlated with the level of serum soluble MHCI chain related molecules (SMIC). SMIC is the ligand of NKG2D, which can activate STAT3, induce MDSCs amplification and M2 polarization of TAMs [107]. When the recruited MDSCs migrated to the tumor area, hypoxia upregulated sialic acid transport and binding to CD45, activated CD45 protein tyrosine phosphorylase, led to rapid dephosphorylation and down-regulation of STAT3 activity, and promoted MDSCs to differentiate into tam in a hif1-independent manner [108]. It seems contradictory for tumor tissues to simultaneously up-regulate and down-regulate the STAT3 activity of myeloid cells, but a dynamic hypothesis can be put forward when considering time and space. When MDSCs permeates into tumor tissue from blood vessels, STAT3 can be up-regulated to amplify MDSCs through the above mechanism. These recruited and amplified MDSCs further infiltrated the tumor tissue, and after entering the deep vascular deficiency area, the hypoxia-driven mechanism first down-regulated STAT3 and promoted the differentiation of MDSCs into TAMs. This positive feedback space-time relationship may be one of the important mechanisms for macrophages and MDSC to promote tumor progression.

### Macrophages and natural killer cells (NKs)

Natural killer cells (NKs), an important component of innate immunity and a class of lymphocytes, are considered to have strong cytotoxic effects on tumor cells. Expression of CD16 and CD56 levels has been used to elucidate two major subsets of NK from humen(NKp46 levels has been used to elucidate a subset of NK from mice), including CD56hiCD16+/-NKs, which secrete inflammatory cytokines, and CD56loCD16hiNKs, which achieve cytotoxic and mediated killing effects [109, 110]. Although the amount of invasion within the tumor is not large, NKs are extremely effective in eliminating malignant cells and limiting tumor metastasis [111]. NKs eliminate tumor cells and limit primary tumor growth by secreting perforin/granulozyme mediated cytotoxicity and by death/apoptosis receptors [109, 112]. Although NK has its unique killing advantage on circulating tumor cells, it has much lower killing efficiency on tumor cells in solid tumor TMEs, which is related to insufficient number and functional inhibition [109, 113]. Thus, both NK subtypes exhibit reduced inflammatory cytokine production and reduced or no cytotoxicity in TME, known as tumor invasive natural killer cells (TINK). For example, studies of pancreatic ductal malignant adenomas (PDAC) showed elevated levels of the NK cell marker CD56 protein in immune cells near PDAC compared to peripheral stroma [113].Also, in non-small cell lung cancer, more NK cells infiltrate the central area of the tumor [114]. However, the absolute number of NK cells is not significant, so the killing effect on tumors is very limited [114]. Although pDC can produce large amounts of type I interferon (IFN) enhanced cytotoxicity of NK cells, the distribution of pDC in non-small cell lung cancer is similar to the distribution of M2 macrophages, so the promotion of NK cells by pDC may not be ideal [114, 115]. In addition, studies based on spontaneous mouse models of breast cancer have found that either M2 TAMs extracted from tumor tissue or TAMs extracted from peritoneum or bone marrow in healthy mice and induced to differentiate in vitro, The cytotoxicity of NK cells can be effectively inhibited by TGFB1-dependent mechanism and the exhaustion of CD27lowCD11bhigh phenotype can be obtained [116]. Although there are few studies on the interaction between NK and macrophages, M2 TAM and NK cells inhibit each other, and M1 TAMs promote NK cell function, which seems to be the result of most current studies [117, 118] (Fig. 1).

### Macrophages and NKT cells

In recent years, more and more studies have been conducted on NKT cells in TME.It is a special type of congenital T lymphocytes with limited CD1d expression, which can be divided into Th1-like, Th2-like, Th17-like, Treg-like, and T-follicle-assisted (TFH) -like NKTs [119]. NKTs also have the same tumor cell killing function as NK cells, but they also switch back and forth between inflammatory and immunosuppressive phenotypes. Among them, iNKT (invariant natural killer T) is currently the main subgroup studied. iNKT cells show innate characteristics and rapidly secrete a large number of Th1 and Th2 cytokines, including interferon-γ, IL-4 and GM-CSF, which may in turn regulate activated APC(antigen-presenting cells) [120–122]. In addition to its different cytokine profiles, iNKT cells also showed strong cytolytic activity, supporting the release of cytotoxic particles containing perforin and granzymes, or activating death receptor pathways involved in the interaction between Fas-FasL and TRAIL-DR5 [123–125].

In order to further clarify the mechanism of immune cell interaction in TME, some articles have gradually emerged in recent years to reveal iNKTs-macrophages crosstalk. The co-localization of NKp46^+^NKT cells with CD163^+^ macrophages reduced the survival of periampulary malignant adenomas, while the co-localization of NKp46^+^NKT cells with CD68^+^ macrophages extended the survival [27]. Unfortunately, in malignant tumor tissues, CD68^+^ macrophages are mostly located in the stroma region away from tumor cells, while NKp46^+^NKT cells are mostly located in the tumor region close to tumor cells, which reduces the opportunity for effective interaction between the two types of cells [27]. The function of NKTs is considerably limited due to the co-localization of macrophages associated with immunosuppression (Fig. 1). Of course, the current research reveals that TAMs is regulated by iNKT cells. For example, iNKT cells mediate M2 TAMs death through the interaction between CD1d and Fas-FasL, while M1 TAMs expressing CD40 is protected from iNKT toxicity [123]. In the pancreatic cancer model, iNKT cells not only have natural anti-tumor effects, but also inhibit M2 TAMs in a microsomal prostaglandin E synthase-1 (mPGES-1) and 5-lipoxygenase (5-LOX)-dependent manner [126]. In contrast, the results obtained in colon adenocarcinoma transgenic mice showed that iNKT cells promoted the M2 polarization of TAMS, increased the expression of FoxP3 protein and the frequency of Tregs, and promoted tumor progression and the formation of intestinal adenomatous polyps [127]. At present, there are few studies on the interaction between NKT cells and TAMs, and there may be great differences in this interaction in different tumors, which needs to be revealed by more studies on the specific mechanism.

## CAR-M replacement strategy for macrophages in TME

**Fig. 2:**
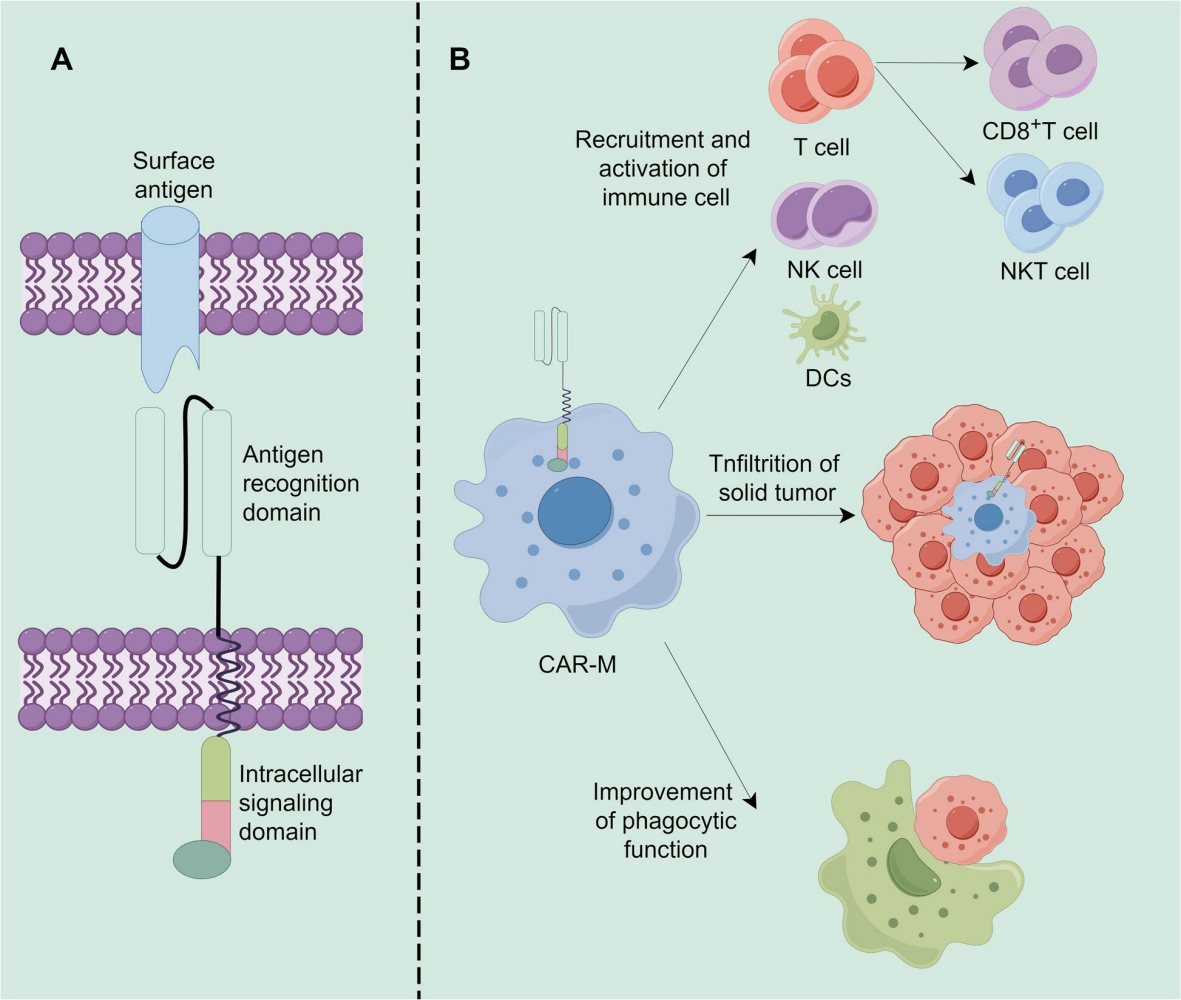
Structure and functions of CAR-macrophages. **A** CAR consists of three domains: antigen recognition domain, transmembrane domain, and intracellular signaling domain. **B** CAR-Macrophages have the ability to recruit and activate T cells, NK cells, and DCs within tumors. They also exhibit enhanced phagocytic capabilities and possess an advantage in infiltrating solid tumors effectively

At present, many reviews have sorted out the therapeutic strategies targeting macrophages [1, 128, 129], which are generally divided into the following ideas: (1) Reduce or eliminate macrophage infiltration in TME; (2) Regulate or restore the anti-tumor function of macrophages in TME; (3) Macrophage function is reprogrammed after transfusion. Among them, the combination of adoptive cell therapy (CAR) of chimeric antigen receptors and macrophages produces CAR-M therapy, which makes up for the shortcomings of insufficient infiltration of tumor tissue by CAR-T and CAR-NK. In mouse models, CAR M increased intratumoral T cell infiltration, NK cell infiltration, dendritic cell infiltration/activation, and TIL activation [130]. In vitro, human CAR-M exhibits antigen-specific phagocytosis, cytokine/chemokine secretion, and kills target antigen-expression targets [131]. CAR macrophages can directly use the CD3ζ intracellular domain and express another kinase Syk containing the tSH2 domain, which binds to CD3ζ and transduces phagocytosis signals in macrophages [132]. Other ITAM(immunoreceptor tyrosine-based activation motif)-containing intracellular domains, such as the γ subunit of the Fc receptor (FcRγ), target EGFR to induce the signature of antibo-dependent cytophagocytosis (ADCP), while a variety of epidermal growth factor-like domain protein 10(Megf10) is associated with macrophage phagocytosis of apoptotic cells [131, 133, 134]. Both can induce phagocytosis similar to CD3ζ. Fortunately, the tandem fusion of CD19pi3k recruitment domain and CAR FcRγ even increased the phagocytosis of target whole cells by three times [133]. The improvement of phagocytic function of CAR-M and the advantages of infiltration of a large number of solid tumors make it have great potential for clinical application. Unfortunately, only a very few Phase I clinical trials (NCT04660929, NCT05007379) have been published. However, CAR-M can be combined with a regimen that depletes macrophages in tumors. First, macrophages in the tumor are depleted to leave a “macrophage blank space” for CAR-M, thus accelerating CAR-M infiltration into the tumor. This combination strategy not only improves the infiltration efficiency of CAR-M, but also reduces the influence of immunosuppressive macrophages on the therapeutic effect after close contact with CAR-M [114]. Meanwhile, CAR-M may also restore tumor killing function of CD8^+^T cells, NK cells and NKT cells (Fig. 2).

## Discussion and prospect

Based on the distribution characteristics of macrophages and the influence of macrophages on other immune cells described in this paper, we propose four possible patterns of spatial distribution of macrophages: 1.Mainly concentrated in the central region of tumor: 2. Mainly concentrated in the peripheral region of tumor;3. Relatively uniform scattered in tumor tissues;4. Cell communities of different sizes are distributed in tumor tissues. Macrophage aggregation in the tumor area appears to be more common in the early stage of the tumor or in the metastases [15, 16]. This may be due to the slow proliferation of tumor cells in the early stage of the tumor, while macrophages have begun to infiltrate around the tumor cells in large numbers. However, the aggregation characteristics in the metastases may be attributed to the contribution of macrophages to the establishment of pre-metastases niches [16, 135, 136]. This concentration of macrophages in the central region of the tumor is ideal for early tumor and metastatic growth, because the relatively high proportion of macrophages in the invasion has more opportunities to block other immune cells. The distribution of macrophages mainly concentrated in the peripheral area of the tumor was more common in advanced and advanced tumors [22, 53]. Immunosuppressive macrophages in this distribution create a pre-invasion niche for tumor cells between tumor tissue and normal tissue, making it easier for tumor cells to invade outward-and harder for other immune cells to penetrate into the tumor for effective killing [114, 137]. Of course, the relatively uniform and rare distribution of macrophages in the tumor tissue may be a good news [15]. Because it may represent that tumor cells have less heterogeneity, it may signal better targeted therapy effect and better prognosis. Conversely, the distribution of macrophages in tumor tissue as communities of varying sizes may represent significant differences in genetic mutations or functional phenotypes among tumor cells in the regions within which these communities reside [101]. This also means that single-gene targeted therapies are difficult to be effective. In addition, these spatial distribution patterns of macrophages may have the potential to be used as prognostic indicators of tumors. For example, the good absorption ability of macrophages to developer enables clinical use of imaging density to judge the infiltration and distribution of macrophages, so as to guide treatment and prognosis [138].

Our previous focus has been on targeted elimination of macrophages, inhibition of macrophage recruitment, and reversal of one of the tumor promoting functions of macrophages, but there are relatively few studies on combining multiple strategies. Therefore, we propose a combination of CAR-M and macrophage exhaustion strategy to improve the efficacy of tumor therapy. Of course, this approach may require coordinated therapies targeting mutations or deletions of p53, which act as a shield that can override the macrophage barrier and promote malignant progression of tumors [14].

## Data Availability

Not applicable.
